# Long-Lasting Latent Neuroschistosomiasis in a Nonendemic Country: A Case Report

**DOI:** 10.7759/cureus.63007

**Published:** 2024-06-24

**Authors:** Janice Alves, Gonçalo V Bonifácio, Rodrigo Vieira, André Militão, Rui Guerreiro

**Affiliations:** 1 Department of Neurology, Unidade Local de Saúde Arrábida, Setúbal, PRT; 2 Department of Neurology, Unidade Local de Saúde do Algarve, Faro, PRT

**Keywords:** myelopathy, infectious myelopathy, neuroschistosomiasis, schistosomiasis, myeloradiculopathy

## Abstract

Neuroschistosomiasis is an uncommon yet serious cause of myelopathy. *Schistosoma mansoni *infection triggers a granulomatous immune response by the human host, resulting in many clinical presentations, depending on the size of the granuloma and its location. The parasitic infection can remain silent for a long period, and this diagnosis should be considered if there is a history of previous exposure in endemic regions. Prompt diagnosis and treatment are crucial for a favorable outcome, minimizing the risk of permanent neurological disability. A case of medullary neuroschistosomiasis is presented, many years after exposure, in a patient who presented with low back pain, rapidly progressing to paraparesis with significant gait impairment. Magnetic resonance imaging findings revealed extensive medullary involvement from the conus all the way to the cervical spine level. After ruling out other causes of myelopathy and considering previous history, total anti-Schistosoma antibodies were tested and detected, confirming the diagnosis. Steroids and schistosomicides were started, with remarkable clinical and imagiological improvement. The patient regained normal muscle strength, gait, and functional independence in the following six months.

## Introduction

Schistosomiasis is the second most common parasitic disease globally [1]. Estimates point toward 200 million people infected, of which 120 million will become symptomatic and about 20 million will progress to a severe form of the disease [2]. It is caused by blood flukes of the genus Schistosoma [3]. *Schistosoma* *mansoni*, *Schistosoma haematobium*, *Schistosoma japonicum*, *Schistosoma mekongi,* and *Schistosoma intercalatum *infect humans [4]. *S. mansoni, S. haematobium*, and *S. japonicum* are most likely to cause clinical manifestations [5].

Humans are infected through contact with contaminated fresh water when motile cercariae attach to and penetrate the skin [5]. During the disease course, the parasite might reach the central nervous system (CNS) [1], causing a granulomatous reaction proportional to the number of eggs that reach the CNS [6]. This phenomenon is described as neuroschistosomiasis [7] and affects an estimated 1%-4% of people with schistosomiasis [2].

*Schistosoma* species have different distributions worldwide, with *S. mansoni* being the most prevalent species in South and Latin America [1], accounting for most cases in Brazil [6].

*S. mansoni* ova are typically larger and more often produce spinal cord lesions [5]. Hence, they can be categorized into three forms: myelopathy or medullary, myeloradiculopathy, and cauda equina syndrome [2].

Medullary neuroschistosomiasis is the most important and frequent form of neuroschistosomiasis. *S. mansoni* and *S. haematobium* are the species most affecting the spinal cord [1]. Clinical presentation and symptom severity depend on the granulomas' location and the intensity of the host's immune response [5]. Spinal root granulomas are associated with radicular and cauda equina symptoms [5] and translate into a typical clinical presentation that usually begins with localized lumbar pain, muscle impairment affecting lower limbs, urinary and sexual dysfunction, and constipation [1]. On physical examination, patients commonly present with paraparesis, diminished or absent patellar and Achilles reflexes (in 79% of patients), and altered sensitivity [1,6].

Schistosomiasis might represent a neglected and underrecognized cause of myelopathy in the clinical setting. Notwithstanding this, even in nonendemic countries, this diagnosis should be considered, especially in younger patients from endemic areas presenting with lumbar pain and bladder dysfunction, evolving to muscle and sensory impairment [5].

## Case presentation

A previously healthy 38-year-old male was admitted due to a recent onset of paraparesis and lower back pain. About three months before admission, the patient developed numbness along the plantar aspect of his left foot, which he attributed to his job as a bus driver, spending long hours seated. In the following week, the condition progressed to numbness in the contralateral foot, followed by rostral dissemination up to the groin. He was started on oral nonsteroidal anti-inflammatory drugs by his family physician, and his symptoms resolved.

A month after the initial episode, he started experiencing lower back pain, which he described as a “pressure,” with an on/off pattern, 6 in 10 intensity, that aggravated when standing and was relieved by lying supine. The following day, the numbness recurred and spread bilaterally from his feet up to his lower abdomen, including the perineal region. Additionally, he described loss of balance and gait difficulties, leading to the inability to walk without assistance in the following weeks. In addition, he developed urinary symptoms such as hesitation and feeling of incomplete micturition, as well as erectile dysfunction.

There was no history of trauma, viral infection, diarrhea, arthralgia, jaundice, abdominal pain, fever, weight loss, or use of any medication, alcohol, tobacco, or recreational drugs. Previous medical history and family history were unremarkable for systemic or neurological disease. The patient was originally from the state of Minas Gerais, Brazil, but had been living in Portugal for the previous four years and had not been back to Brazil or traveled elsewhere since. When questioned, he revealed that, while in Brazil, he had frequent exposure to fresh water throughout his childhood up until his early 20s.

**Figure 1:**
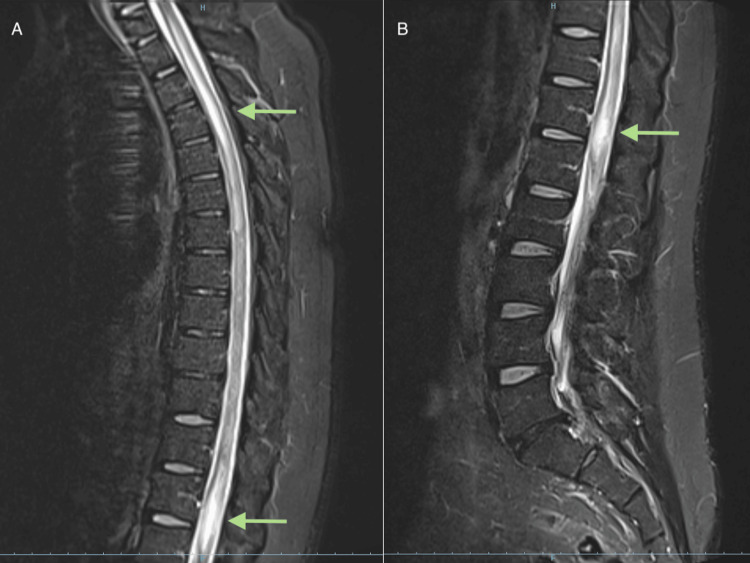
Initial (A) dorsal and (B) lumbar spine MRI images, showing hyperintensities in sagittal short-tau inversion recovery sequence (green arrows) MRI: magnetic resonance imaging

After multiple visits to the emergency department, dorsal and lumbar magnetic resonance imaging (MRI) was obtained. The MRI revealed concentric medullary expansion between the conus medullaris and D7-8, as illustrated in Figure 1, with multifocal heterogeneous enhancement. This suggests a primary tumor vs. an inflammatory vs. infectious lesion, and a Neurology consult was requested.

The initial examination was remarkable for decreased strength in both lower limbs, grade three on the Medical Research Council scale, with absent knee and ankle jerks on the left and reduced on the right. There was a reduced sensation to pinprick bilaterally all the way up to the D10 level and a reduced vibratory sensation in both toes. Additionally, in the heel-to-shin test, the patient presented ataxia in both lower limbs and could not walk without bilateral support.

The complete blood count, urea nitrogen, electrolytes, and vitamin B12 levels were normal. The cerebrospinal fluid (CSF) analysis revealed pleocytosis (65 white blood cells/µL), predominantly lymphocytic (90%), and elevated protein content (143 mg/dL). CSF polymerase chain reaction for herpes simplex, cytomegalovirus, Epstein-Barr virus, human T-lymphotropic virus (HTLV)-1, enterovirus, and parechovirus were negative. The bacteriologic cultures and venereal disease research laboratory tests were also negative. Blood serology for human immunodeficiency virus and HTLV-1/2 were also negative.

Additionally, the cranial MRI was unremarkable, while the cervical MRI revealed medullary hyperintensity in T2 and short-tau inversion recovery from C5-C6 along the entire spinal cord associated with medullary expansion.

Furthermore, the CSF analysis showed no neoplastic cells or oligoclonal bands. Additionally, autoantibodies against aquaporin 4 and myelin oligodendrocyte glycoprotein were also absent. Fecal parasitology for parasites or Schistosoma eggs was negative, and *S. haematobium* eggs were not found in urine samples. However, total anti-Schistosoma antibodies were positive at 1/320 (cutoff ≥1/160).

The patient was started on intravenous methylprednisolone (1 g/day for five days). Schistosomal myelopathy diagnosis was presumed, and the patient was treated with praziquantel (a single dose of 50 mg/kg) and prednisolone (1 mg/kg/day for six months), and an intensive rehabilitation program was initiated. Three months later, he experienced significant clinical improvement. He reported less pain and numbness along his lower limbs, marked strength improvement (score four on MRC), and mild ataxia, and could walk unaided. By this time, the MRI revealed some expansion but hypersignal reduction from C5 all the way to the conus medullaris.

**Figure 2:**
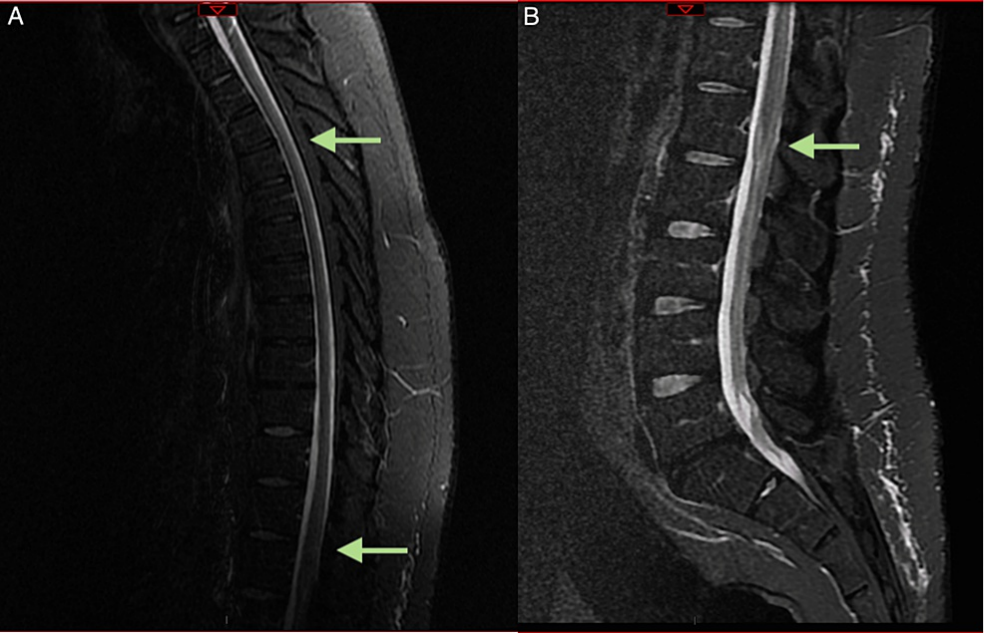
(A) Dorsal and (B) lumbar spine MRI images six months after treatment, showing imagiological improvement in sagittal short-tau inversion recovery sequence (green arrows) MRI: magnetic resonance imaging

The patient returned to work approximately six months later and resumed his normal daily life. He reported ongoing pain and numbness on the anterior aspect of his thighs, mild urinary urgency, and erectile dysfunction. Neurological examination revealed normal muscle strength and deep tendon reflexes, slight hypopallesthesia on both toes, and mild ataxia in both lower limbs. However, he was able to walk independently with a normal gait. MRI revealed a normal cervical and conus medullaris T2 signal, persisting a slight hypersignal between D8 and D10-D11, as shown in Figure 2.

## Discussion

We present the case of a Brazilian patient, originally from a state endemic to *S. mansoni*, with extensive schistosomal myelopathy after a long latency of at least four years, with a remarkable clinical and imagiological recovery after steroid and praziquantel treatment. In this case, the diagnosis was made based on epidemiological context and evidence of exposure, suggestive clinical presentation, typical MRI findings, positive serological test for Schistosoma [8], exclusion of other causes of transverse myelitis, and response to therapy.

In nonendemic areas, we do not tend to exclude diagnoses involving a rare tropical infection [9]. It becomes especially challenging when it is not associated with a recent exposure, as in this patient. This may lead to a longer time to reach a diagnosis and potentially delay treatment. We found this case particularly remarkable because of its prolonged latency, with an unusual presentation of extensive myelitis, involving the cervical and dorsal cord and the conus medullaris.

In fact, neurological forms of schistosomal infection are rare, even in endemic areas, and early diagnosis is crucial for prompt treatment, enabling full recovery of neurological deficits [1,9]. In nonendemic regions, neuroschistosomiasis is even more infrequent. Thus, the diagnosis might be even further delayed and particularly challenging [1].

Epidemiologically, it occurs more often in men from rural areas, probably because they are more prone to contact with infected water [8], as also reflected in our patient’s background. According to Palin et al. [8], men are more likely to engage in heavier physical activity, facilitating the migration of eggs and, therefore, increasing the likelihood of infestation.

Regarding the medullary level, most lesions affect the lower thoracic spine, around 40%, followed by the lumbar (15.8%) and the lumbosacral region (0.7%) [1]. Cervical involvement is rather uncommon, with only a few cases reported [10]. This distribution is explained by the anastomotic veins between Batson’s venous plexus and pelvic veins, connecting the portal system and the inferior vena cava to the avalvular veins of the spinal canal [1]. This route allows the migrations of eggs and adult worms [1], triggering an immune response from the host. Clinical presentation will depend on the affected region, number of eggs deposited, and subsequent intensity of the inflammatory response [1], hence translating into multiple clinical presentations with a wide range of nonspecific symptoms and signs, making the diagnostic process even more challenging. Diagnosis is mostly presumptive and based on clinical features and test results, including neurological symptoms and/or lesions of a corresponding spinal cord level, biochemical and immunological analysis, epidemiological and parasitological or serological confirmation of schistosome infection, and exclusion of other causes of transverse myelitis [3,6].

Concerning laboratory analysis, in most cases, CSF examination reveals pleocytosis (91%), with a predominance of lymphomononuclear cells and a discrete or moderate increase in protein levels (95%), along with normal glucose levels [1]. As for parasitological examination, the characteristic ova can be identified in stool in around 40% of cases [5].

Although MRI findings are nonspecific, they can support the diagnosis and exclude other differentials. MRI is the most sensitive method, and the usual findings encompass spinal cord enlargement, nerve root thickening, and intramedullary T2 hyperintensity with T1 hypointense signal, highlighting the associated edema [2]. A patchy pattern of contrast uptake is observed in T1-weighted images, reflecting a nodular pattern corresponding to granulomas [1].

The presence of the MRI abnormalities supports the diagnosis, expediting treatment and avoiding invasive procedures [11]. Ultimately, the diagnosis is still frequently presumptive, and treatment should be considered since it will usually lead to a good clinical response.

Treatment comprises a combination of schistosomicides (which destroy adult worms by interrupting egg production and preventing the inflammatory reaction of the nervous tissue), corticosteroids, and rehabilitation [1]. Praziquantel is the primary antihelminthic drug of choice [1], with a reported cure rate of 70%-90% [2] in a single dose of 50 mg/kg in adults [1]. Corticosteroids are usually recommended before praziquantel administration to control the inflammatory response, as they reduce inflammatory activity, compression, and destruction of nerve tissue. An initial course of five days of intravenous methylprednisolone pulse therapy can be given at the dose of 15 mg/kg/day (maximum daily dose of 1 g), followed by a maintenance dose of prednisolone (1 mg/kg) [1]. There is no consensus regarding the ideal corticosteroid treatment duration. However, there is some evidence that withdrawal before six months of treatment might increase the risk of relapse [1].

In a case series by de Wilton et al. [2], four cases of neuroschistosomiasis were described, and the mean time between possible exposure and symptom onset was 12.7 months (0-29 months). All subjects presented with back pain, weakness in the lower limbs, altered sensation, and urinary dysfunction. CSF lymphocytosis was present in all four patients. A parasitological examination for ova detection in stool or urine was negative in all patients. Regarding MRI findings, three patients presented typical lower thoracic spine enhancement, and one subject with conus enhancement. All subjects were treated with praziquantel (minimum of three days) and a high dose of corticosteroids. Only one out of the four patients fully recovered.

Although neuroschistosomiasis is a rather uncommon complication of schistosomiasis, the presumptive diagnosis should be considered. Despite its possibly clinically severe presentation, prompt and adequate treatment is associated with an excellent prognosis [8].

## Conclusions

Although schistosomiasis is not endemic to Portugal, we must consider alternative diagnoses in patients with relevant epidemiological context in a globalized world, highlighting the importance of a thorough history and examination. Furthermore, the increased latency of clinical manifestations and extensive medullary involvement seen in this case were also remarkable features, making this case even more noteworthy.

Recommendation

Neuroschistosomiasis still represents an important public health problem and its presumptive diagnosis should not be neglected, since prompt diagnosis and treatment are crucial for a favorable outcome, minimizing the risk of permanent neurological disability.

## References

[1] Dastoli PA, Leite AL, da Costa MD, Nicácio JM, Pinho RS, Ferrarini MA, Cavalheiro S (2021). Medullary neuroschistosomiasis in adolescence: case report and literature review. Childs Nerv Syst.

[2] de Wilton A, Aggarwal D, Jäger HR, Manji H, Chiodini PL (2021). Delayed diagnosis of spinal cord schistosomiasis in a non-endemic country: a tertiary referral centre experience. PLoS Negl Trop Dis.

[3] Da Silva MA, Nai GA, Tashima NT (2019). Schistosomal myeloradiculopathy - a case report. Rev Soc Bras Med Trop.

[4] Ferrari TCA, Moreira PRR (2011). Neuroschistosomiasis: clinical symptoms and pathogenesis. Lancet Neurol.

[5] Thakur K, Zunt J (2015). Tropical neuroinfectious diseases. Continuum (Minneap Minn).

[6] Domingues ALC, Barbosa CS, Agt TFA (2020). Spinal neuroschistosomiasis caused by Schistoma mansoni: cases reported in two brothers. BMC Infect Dis.

[7] Rose MF, Zimmerman EE, Hsu L (2014). Atypical presentation of cerebral schistosomiasis four years after exposure to Schistosoma mansoni. Epilepsy Behav Case Rep.

[8] Palin MS, Mathew R, Towns G (2015). Spinal neuroschistosomiasis. Br J Neurosurg.

[9] Szekeres C, Galletout P, Jaureguiberry S, Crickx E, Monsel G, Chabriat H, Jouvent E (2013). Neurological presentation of schistosomiasis. Lancet.

[10] Carod Artal FJ (2012). Cerebral and spinal schistosomiasis. Curr Neurol Neurosci Rep.

[11] Mikulich O, Chaila E, Crotty JM, Watts M (2013). Spinal cord schistosomiasis presenting as a spinal cord syndrome. BMJ Case Rep.

